# Using a Module-Based Analysis Framework for Investigating Muscle Coordination during Walking in Individuals Poststroke: A Literature Review and Synthesis

**DOI:** 10.1155/2018/3795754

**Published:** 2018-06-03

**Authors:** Bryant A. Seamon, Richard R. Neptune, Steven A. Kautz

**Affiliations:** ^1^Ralph H. Johnson VA Medical Center, Charleston, SC, USA; ^2^Department of Health Sciences and Research, College of Health Professions, Medical University of South Carolina, Charleston, SC, USA; ^3^Department of Mechanical Engineering, The University of Texas at Austin, Austin, TX, USA; ^4^Division of Physical Therapy, College of Health Professions, Medical University of South Carolina, Charleston, SC, USA

## Abstract

Factorization methods quantitatively group electromyographic signals from several muscles during dynamic tasks into multiple modules where each module consists of muscles that are coactive during the movement. Module-based analyses may provide an analytical framework for testing theories of poststroke motor control recovery based on one's ability to move independently from mass flexion-extension muscle group coactivation. Such a framework may be useful for understanding the causality between underlying neural impairments, biomechanical function, and walking performance in individuals poststroke. Our aim is to synthesize current evidence regarding the relationships between modules, gait mechanics, and rehabilitation in individuals poststroke. We synthesized eleven studies that performed module-based analyses during walking tasks for individuals poststroke. Modules were primarily identified by nonnegative matrix factorization, and fewer modules correlated with poor walking performance on biomechanical and clinical measures. Fewer modules indicated reduced ability to control individual muscle timing during paretic leg stance. There was evidence that rehabilitation can lead to the use of more and/or better-timed modules. While future work will need to establish the ability of modules to identify impairment mechanisms, they appear to offer a promising analytical approach for evaluating motor control.

## 1. Introduction

Walking is a complex locomotor task made up of biomechanical functions that require well-coordinated muscle activity [1]. Muscle coordination, the ability to control the activation and timing of multiple muscles, allows individuals to maintain a high level of movement complexity and safety while overcoming task variability that can occur in the environment during dynamic tasks, such as walking [2]. The neurologic damage resulting from stroke in severe cases will often result in paralysis of the contralesional (paretic) leg or a reliance on mass limb flexion-extension patterns of muscle activity, instead of the usual coordinated activity throughout the gait cycle. Therefore, a prominent clinical theory of motor control recovery in stroke is based on one progressively regaining the ability to move independent of mass limb flexion-extension activation thereby increasing the capacity for more complex movements [3, 4]. There is a rich history of clinical observation and research to support this theory; however, many of the analysis-based frameworks or assessment tools for answering questions related to motor control recovery are based on observational ratings or evaluated in isolated movements such as the Fugl-Meyer [3, 5–8]. Unfortunately, assessments that evaluate motor control in single-limb isolated movements, like the Fugl-Meyer, are limited in their ability to serve as functional assessments for quantifying motor control in dynamic mobility tasks. Thus, there is a need for an analysis framework that can quantify levels of muscle coactivation during dynamic tasks, such as walking. Such a framework may be useful for understanding the causality between underlying neural impairments, biomechanical function, and walking performance in individuals poststroke. Answering these gaps in understanding can provide researchers and clinicians a theoretical basis for selecting or developing targeted interventions aimed at improving rehabilitation outcomes for individuals poststroke [9]. Recently, a method for drawing conclusions between neural activity and biomechanics has been to assess muscle activity during dynamic tasks using electromyography (EMG) from multiple muscles and applying mathematical techniques such as nonnegative matrix factorization (NNMF) or principal component analysis (PCA). These factorization methods allow the grouping of muscles into modules based on their EMG amplitude and timing during specific portions of a given task [10, 11]. Therefore, a module can be defined as a group of muscles that are coactivated and share the same time course of activation during a task.

A module-based analysis was first applied to human walking by Ivanenko et al. [12]. They identified 5 separate modules, which remained stable as groupings of coactive muscles used to accomplish walking across multiple factorization methods even with changes in walking speed, body weight support, or adding an additional task during gait [12, 13]. Similarly, Clark et al. [14] identified 4 modules in healthy controls that compared favorably with those identified by Ivanenko et al. [12] despite only using lower extremity muscle EMG. Module consistency across multiple walking conditions and investigators [13] led to the idea that modules may provide a low-dimensionality view of neuromechanical output and a way of quantifying locomotor complexity (with less modules indicating a lower complexity of movement) for individuals poststroke, especially if modules can be used to explain biomechanical function.

**Figure 1:**
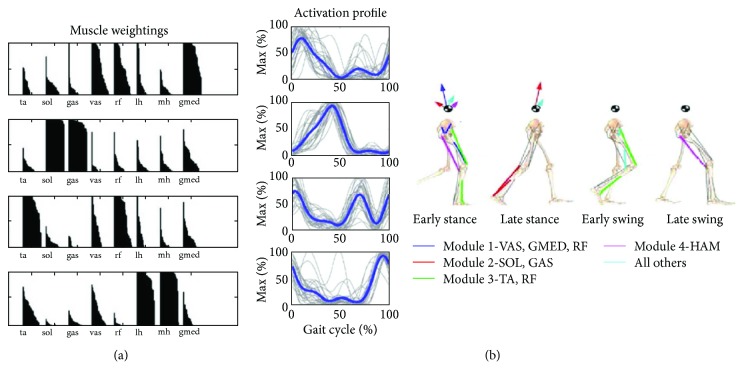
Image and caption modified from Clark et al. [14] and Neptune et al. [15]. (a) Muscle coactivation weightings from healthy individual walking at 1.2 m/s determined from NNMF. (b) Activation profiles represent the timing of the module during the gait cycle. The thin lines were individuals from Clark et al. [14] with thicker lines representing the group average. Module contributions to walking are demonstrated on the skeleton rendition. Arrows are acting on the center of mass symbol to illustrate what the module contributions are to ground reaction forces for propulsion during walking. Module 1 can be seen to provide body support and decelerate forward motion. Module 2 contributes to body support as well but provides forward propulsion. Module 3 assists with limb clearance during swing phase and module 4 with limb deceleration. TA: tibialis anterior; SO: soleus; MG: medial gastrocnemius; VM: vastus medialis; RF: rectus femoris; MH: medial hamstrings; LH or HL: lateral hamstrings; GM: gluteus medius.

Neptune et al. [15] and McGowan et al. [16] investigated the relationship of modules with biomechanical functions of walking. They were able to relate each module (including a 5th module, which would was predicited to include iliopsoas activity even though EMG was not collected from this muscle) to a primary biomechanical function during walking using module data from healthy individuals studied by Clark et al. [14]. Modules 1–4 and their biomechanical functions are identified in Figure 1 [15, 16]. During early stance, module 1, primarily made up of extensor (gluteus medius and quadriceps) activity, contributed to body support while module 2, primarily made up of plantar flexor activity, contributed to body support and propulsion. During early and late swing, module 3, primarily made up of rectus femoris and ankle dorsiflexor activity, acted to decelerate the leg in the early and late swing. Module 4, primarily made up of hamstring activity, allows for a coordinated transition from flexion to extension in order to support limb deceleration in mid to late swing and trunk propulsion in early stance.

The ability of a module to quantitatively represent common coactivation patterns of muscles during walking, and the fact that they perform specific biomechanical functions, makes module-based analysis an appealing method to measure recovery of motor control in individuals poststroke. This is especially true given that a quantitative assessment of motor control during mobility tasks could provide a more accurate assessment of functional ability for individuals poststroke than clinical measures such as the Fugl-Meyer. Researchers and clinicians would have a measure to accurately assess control during a dynamic task and monitor treatment progress from interventions designed to address muscle coordination. Since individuals poststroke commonly exhibit varying degrees of ability to move from a mass flexion-extension coactivation of muscles to more independent control commonly seen in normal walking, we would expect those with mass coactivation during the gait cycle to have fewer modules (perhaps 2) and those with greater control to have more modules (perhaps 3–5). Under this framework, measures of module number, composition, and control are all variables which might be expected to improve with recovery. These variables may be able to describe observed biomechanical alterations in poststroke walking and thus may yield insights into underlying impairments and bring to light why some treatments are more effective for specific patient subgroups [17]. This would aid in rehabilitation treatment precision and presumably result in more robust outcomes. Overall, module-based analyses have the underpinnings to be a powerful quantitative tool for understanding locomotor complexity poststroke by providing the ability to quantify muscle coordination with respect to independence from a mass flexion-extension coactivation.

Despite the potential for modules to quantify muscle coordination during walking, there is not a well-agreed-upon method for determining them in individuals poststroke. Similarly, there is little consensus on the relationship between modules and gait mechanics or function in individuals poststroke or how they may change with rehabilitation. Overall, there is a direct need to determine the potential for a module-based motor control framework to answer important research and clinical questions related to stroke motor control recovery. To address this need, we aim to synthesize the current evidence, much of which is from our research group, regarding the relationship between modules and gait mechanics, rehabilitation outcome measures, and their response to intervention in individuals poststroke during walking.

## 2. Methods

### 2.1. Overview

We conducted a systematic review protocol based on the Preferred Reporting Items for Systematic Review and Meta-Analysis (PRISMA) statement guidelines [18] in order to report on the current state of the literature, in addition to our own research, on performing module-based analysis of coordination during gait and functional outcomes in the poststroke population.

### 2.2. Search Strategy

We systematically searched the following electronic databases: MEDLINE (via PubMed), Scopus, CINAHL, PEDro, and OT Seeker. The search was limited from January 2007 to April 2018 and only included studies published in English with human subjects. A variety of terms and MESH headings were selected with the assistance of a reference librarian at the Medical University of South Carolina using keywords agreed upon by the authorship team and drawn from publications examining factorization of EMG. The full search string for each database can be found in Table 1. Additional articles were identified by reviewing the reference lists of the included citations. All citations were uploaded into EndNote x8 to remove duplicates.

### 2.3. Selection Criteria

All studies were considered eligible in which participants had suffered a stroke and were greater than 18 years of age. Studies with mixed populations were included if data was available for a stroke subgroup. Studies were included that contained a method of factorization for analyzing EMG of lower extremity muscles during gait and referenced variables with functional relevance such as joint kinematics, gait speed, or clinical exams. Conference proceedings and dissertations were excluded.

### 2.4. Study Selection

All article titles and abstracts were screened for inclusion independently by one author (BS). A second author (SK) reviewed 25% of the references to establish validity of the study selection criteria. When a title or abstract was insufficient to determine if a study met the inclusion criteria, the full paper was reviewed.

### 2.5. Method of Quality Assessment

Each study's quality was assessed by the lead author using a modified Downs and Black Checklist. The original checklist contains 27 items that evaluate five categories including reporting quality, internal and external validity, bias, and statistical power. The checklist has strong test-retest reliability (*r* = 0.79), interrater reliability (*r* = 0.75), and internal consistency (KR-20 = 0.88) [19]. There is a precedent for modifying the checklist by excluding the 12 items related to interventional studies; however, it is unknown how this modification affected the checklist's reliability [20–22]. For the purpose of this review, we decided to use the previously published modified version due to the limited number of intervention studies included. Each article could receive a maximum score of 16. We converted our raw scores into percentages and divided them into categorical levels of quality (“good,” “fair,” and “poor”) to help with interpretation [23]. This decision is consistent with the previous use of the modified Downs and Black Checklist where appraisal scores of 71% or greater were labeled as “good,” 54–70% as “fair,” and 53% or less as “poor” quality [24].

### 2.6. Data Extraction

A single author (BS) extracted the following data from each article: study characteristics, participant demographics, gait or mobility tasks, EMG protocol, method of factorization, statistical approach, and functionally relevant variables. These results were reviewed by a second author (SK) for accuracy.

## 3. Results

### 3.1. Literature Search

**Figure 2:**
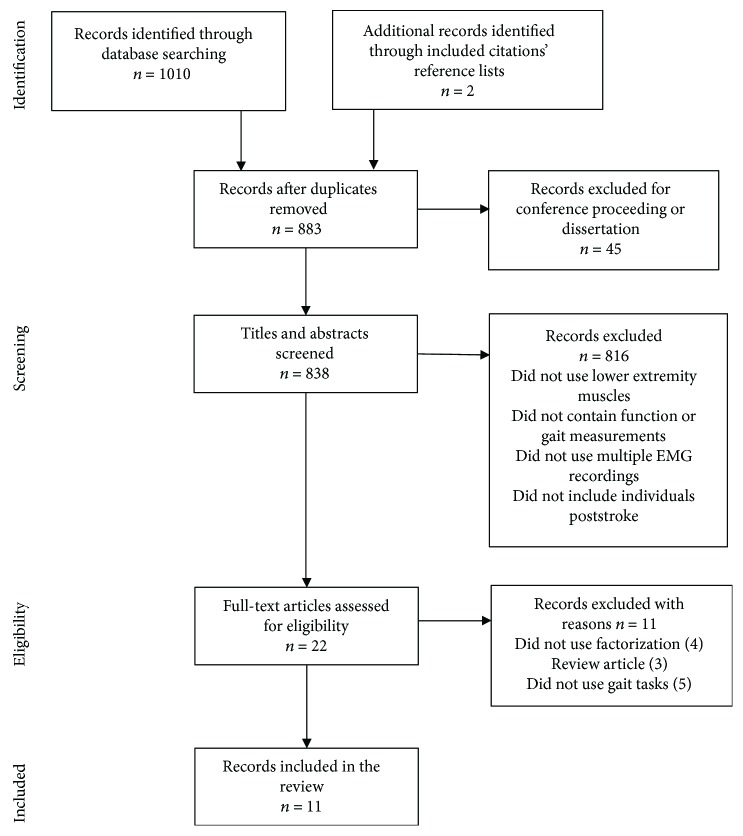
Search results.

Our search of electronic databases returned 1010 articles. Two additional articles were identified from a review of included titles' reference lists. Using our inclusion and exclusion criteria, 22 articles were selected for full text review based on screening titles and abstracts. A total of 11 articles met the final inclusion for review. The results of our search can be found in Figure 2 including reasons for excluding articles. Details of these studies are presented in Tables 2–4. As expected, the majority of included studies (6) were conducted by individuals from our lab group and reported results using the same sample population. These studies have been noted accordingly in Table 2.

### 3.2. Study Quality

Results of methodologic quality using the modified Downs and Black Checklist [22] are presented in Table 5. Out of the 11 reviewed articles, 7 were found to have good evidence quality based on a percentage score greater than 71% [23, 24].

### 3.3. Muscles Used in Module-Based Analyses

EMG was recorded from a variety of muscles during walking tasks in both healthy individuals and those poststroke [14, 25–34]. In addition to steady state walking, one paper had subjects perform variations of gait mechanics through adjusting cadence, step height, and length until a steady-state was reached to compare EMG between normal and altered mechanics [33]. Another study compared EMG recordings from over ground and treadmill walking at self-selected speeds during steady state walking [31]. The number of muscles included in analyses varied from 32 [29] to 7 [28]. All studies collected EMG data from lower extremity muscles [14, 25–27, 29–34] with two papers additionally including muscles from the upper extremity and trunk [29, 34]. Common lower extremity muscle groups included the following: tibialis anterior, soleus, at least one head of the gastrocnemius, rectus femoris, either vastus medialis or vastus lateralis, one of the hamstring muscles, and either gluteus maximus or gluteus medius [14, 25–27, 29–33]. The 8-muscle set of the tibialis anterior, soleus, medial gastrocnemius, rectus femoris, vastus medialis, medial and lateral hamstrings, and gluteus medius was consistently used in 7 of the 11 studies and had the strongest level of collective evidence [14, 25, 26, 31–33].

### 3.4. Module-Based Analysis Methods

The most common factorization technique for determining modules was nonnegative matrix factorization (NNMF). Ten of the 11 articles evaluated used NNMF to group lower extremity muscle EMG activity normalized to the gait cycle into modules [14, 25, 26, 28–34]. Principal component analysis was the other method reported [27]. All articles reviewed preselected a threshold criterion for selecting the number of modules using factorization. Each of the paper's methodologies was applied to healthy individuals for comparison with participants poststroke. The majority of studies using NNMF [14, 25, 26, 28, 31–34] employed methods originally described by our group in Clark et al. [14]. This method defines the number of modules where 90% of the variance is accounted for during the six phases of gait and for each muscle's activity during the gait cycle. In order to increase the number of modules, an additional module must increase the variance explained of the lowest percentage by at least 5 percent [14]. Using this method, the consistent number of identified modules in healthy individuals was 4 [14, 25, 26, 28, 31, 33] with a small subset of participants requiring 5 [14]. Hashiguchi et al. [30] reported module assignment with this method to have a high test-retest reliability (ICC = 0.81). In comparison, Gizzi et al. [29] used NNMF for module assignment with an 80% threshold for the percentage of variance accounted for. Despite a lower threshold, they also identified 4 modules in healthy individuals when examining upper extremity, trunk, and lower extremity muscles together as well as during their reanalysis using only lower extremity muscles.

**Figure 3:**
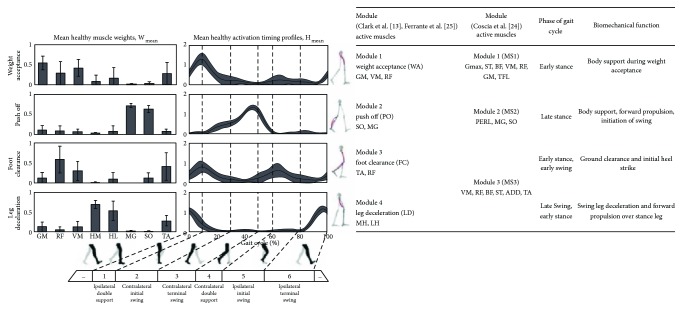
Comparison of healthy modules across included studies. Common modules found during normal steady state walking as described for comparison by Clark et al. [14], Ferrante et al. [28], and Coscia et al. [27]. For each module, the primary muscle activity which composes the module is listed as well as the corresponding phase of gait and biomechanical function. The image reproduced from Ferrante et al. [28] demonstrates the muscle weightings and timing for each module and highlights the phase of the gait cycle where each module is used. Key coactive muscles in each module are highlighted red on the lower body illustration. TA: tibialis anterior; SO: soleus; MG: medial gastrocnemius; VM: vastus medialis; RF: rectus femoris; MH: medial hamstrings; LH or HL: lateral hamstrings; GM: gluteus medius.

In contrast to NNMF methods, Coscia et al. [27] used principal component analysis with preselected threshold *eigenvalues* > 0.5 and >1.0 for module assignment. A threshold of *eigenvalues* > 0.5 resulted in 5 modules for healthy individuals in order to account for 85% of the variance. The authors adjusted their threshold to *eigenvalues* > 1.0 in order to obtain a consistent number of modules across healthy individuals and those poststroke. This change resulted in 3 modules, which accounted for 75% of the variance explained in the muscle activity throughout the gait cycle for individuals who were healthy and poststroke. Coscia et al. [27] named the modules MS1, MS2, and MS3. Modules MS1 and MS2 represented similar phases of gait with the modules 1 and 2 presented in both Clark et al. [14] and Ferrante et al. [28], while MS3 is reported as a grouping of the modules 3 and 4. A detailed description of the modules from each of these three author groups is presented in Figure 3 in a side by side comparison with a visual representation produced by Ferrante et al. [28].

### 3.5. Module Differences in Individuals Poststroke

#### 3.5.1. Common Module Merging Patterns Poststroke

**Figure 4:**
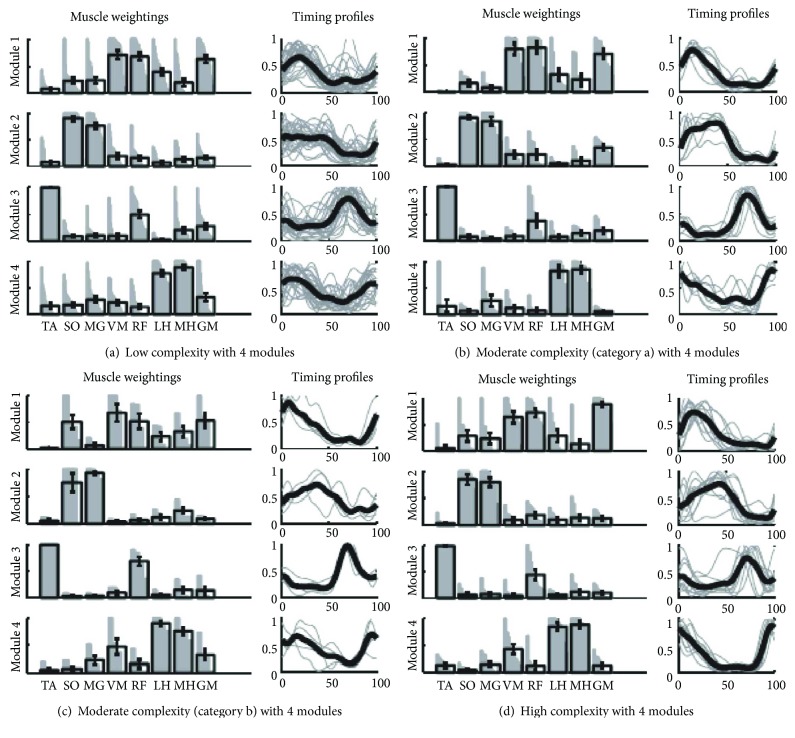
Image and caption reproduced from Clark et al. [14]. Module muscle weightings and activation timing profiles identified when NNMF was performed using 4 modules in all paretic legs. Refer to Figure 3 for the meaning of gray and black bars and lines. Associations within each group for muscle weightings and activation timing profiles are quantified in Table 3, (c) and (d), respectively. (a) The low complexity subgroup had modules with independent composition (muscle weightings) but similar timing of modules 1, 2, and 4. (b) The category A moderate complexity subgroup had modules with independent composition but similar timing of modules 1 and 2. (c) The category B moderate complexity subgroup had modules with independent composition but similar timing of modules 1 and 4. (d) The high complexity subgroup had modules with independent composition and activation timing profiles that were less correlated than in the moderate and low complexity subgroups [14].

The reviewed studies found the paretic leg of individuals poststroke often used 2 or 3 modules, instead of 4 or 5, when walking [14, 25–34]. In individuals with 2 modules, there is strong cocontraction of flexor or extensor muscle groups. Clark et al. [14] labeled these as a stance module and swing module. Clark et al. [14], Allen et al. [25], and Ferrante et al. [28] found that when individuals poststroke express 3 modules then common merging patterns surface. Two common merging patterns, categories A and B, were identified by Clark et al. [14] and Allen et al. [25] and are visually represented in Figure 4. Category A is a merging of modules 1 and 2, while category B is the merging of modules 1 and 4. Category A module merging results in reduced propulsion during push off due to increased activation of the hamstrings in late stance phase. Category B results in increased hamstring activity during mid stance and late stance resulting in more knee flexion rather than hip extension. Category B individuals also had decreased propulsion thought to be due to premature plantarflexion of the ankle in mid to late stance phases as a compensation for the reduced body support caused by increased knee flexion during stance [14, 25]. Ferrante et al. [28] reported merging patterns as well. One subject's modules were similar with category B; however, their second subject had a new merging pattern that consisted of modules 3 and 4. Clark et al. [14] proposed the number of modules be defined as representing one's locomotor complexity, with fewer modules indicating less independently timed control of muscle activity and hence lower locomotor complexity.

#### 3.5.2. Module Composition and Control

In addition to module number, three papers evaluated if the composition (represented by the weighting of individual muscles) and control (represented by a timing curve) could be used to separate healthy and poststroke walking [27, 29, 32]. All three suggest that impairments in composition and control variables are present in individuals poststroke in the paretic and may also exist in the nonparetic leg [27, 29, 32]. Specifically, Gizzi et al. [29] reported differences in module composition and control between controls and both the paretic and nonparetic legs of individuals poststroke during walking. Routson et al. [32] found changes in the ability to control module 2 between healthy controls and individuals poststroke who used 4 modules. Additionally, Coscia et al. [27] reported that speed influenced the amplitude of EMG activity in modules 1 and 2, which could influence module composition between an individual poststroke's paretic and nonparetic legs. Although, two studies reported that speed does not impact overall module number in healthy individuals [29, 32]; the finding of Coscia et al. [27] may provide evidence that changing speed can impact the composition of a module or shed light on the influence of task difficulty on muscle coordination present poststroke [33]. In addition, one study by Kautz et al. [31] found that module assignments were the same regardless of over ground or treadmill walking at self-selected speeds.

### 3.6. Poststroke Module Relationships with Gait and Rehabilitation Outcome Measures

#### 3.6.1. Gait Mechanics

There is strong evidence that a reduced number of modules during walking poststroke correlates with a reduction in gait speed [14, 26, 32–34]. The reduction in modules does not appear to be speed dependent as walking slower than a self-selected speed did not change the number of modules in healthy individuals [12, 27]. Fewer modules are correlated with impaired paretic propulsion [14, 25, 26, 32, 33], reduced mediolateral stability during stance [25], reduced paretic step ratio [14, 26], and increased time in paretic preswing [14, 25, 26]. The ability to change speeds, cadence, step height, and step length during steady-state walking is also decreased in individuals having fewer modules poststroke [33].

#### 3.6.2. Functional Ability

There is limited evidence in regard to the relationship between the number of modules for a given individual and their level of independence as measured by rehabilitation outcome measures. Three of the 11 papers reported on clinical measures of functional ability commonly used in rehabilitation and modules. However, only one paper, Bowden et al. [26], provided correlation data between the two. In their cross-sectional design, Bowden et al. [26] reported a higher correlation between the number of modules and scores on the Berg Balance Scale and Dynamic Gait Index than with the Fugl-Meyer lower extremity scale.

### 3.7. Poststroke Module Responses to Rehabilitation Interventions

One study used a prepost intervention design to assess the impact of standard rehabilitation care on module use. Hashiguchi et al. [30] assessed module number during hemiparetic walking prior to participants completing 1 month of inpatient rehabilitation. Participants demonstrated improvements in rehabilitation outcome measures including the Barthel Index, Berg Balance Scale, Timed Up and Go, gait speed, and paretic lower extremity strength without an accompanying increase or decrease in the number of modules used during gait [30]. The authors sought to quantify the amount of merging or fractionation of each module used by modifying the merging index and fractionation index as described by Cheung et al. [35] which have been used in evaluating upper extremity reaching tasks. The authors did not correlate these indices to biomechanical function, which limits conclusions on their physiological relevance at this time.

Two studies assessed module changes in response to targeted therapies for improving muscle coordination [28, 32]. Ferrante et al. [28] applied functional electric stimulation (FES) during supported treadmill walking. The FES evoked muscle activation that mimics healthy module composition and control. Participants increased the number of modules used from 3 to 4 and reported improvements on a global rating change scale. The participants also improved scores on the Mini Best test; however, they did not improve Fugl-Meyer scores [28]. Routson et al. [32] reported on a subset of participants who completed 12 weeks of body weight, supported treadmill training, and gained the ability to use 4 modules. They investigated whether these individuals had changes in control of module use by evaluating module timing curves after training. Those who had 3 or 4 modules pretherapy demonstrated an improvement in timing of module 2, which is primarily responsible for limb propulsion during push off [32]. Routson et al. [32] reported a similar finding for individuals who had 2 modules pretraining; however, this trend did not reach significance.

## 4. Discussion

This review employed PRISMA systematic review methodology to synthesize current evidence for using a module-based analysis of muscle coordination during poststroke walking while report on module relationships with gait mechanics, rehabilitation outcome measures, and response to intervention. We found that the majority of the evidence in this area has been conducted by our group. Nevertheless, we found that there is a general agreement that NNMF of EMG signal observed from a common set of eight leg muscles during steady-state walking results in 4 modules for healthy individuals. For individuals poststroke, the most severely affected subjects often have only two modules in the paretic leg, representing a mass flexion and extension pattern of muscle activity during walking. As individuals poststroke show more independence from a mass flexion and extension pattern (i.e., increased number of modules with composition and timing that mimic healthy individuals), their walking performance improves. Thus, there is apparent support for module-based analyses to develop a motor control framework based on independence from mass flexion-extension patterns.

As is expected, modules resulting from EMG decomposition are dependent upon the method of linear decomposition and the threshold in variance accounted for VAF used to decide their number [36–38]. The primary example of this in our review is Coscia et al. [27] who set a 75% VAF threshold and found the number of modules observed during gait was 3 compared to 4 or 5 as determined using the more conventional 90% VAF threshold [12, 14, 28, 30, 34]. In addition, other examples in the literature have demonstrated how altering factorization methods, EMG data structure, or VAF can cause changes in module construction [36–38]. While there may be research questions that can be answered when VAF is less than 90% or other methodological considerations are altered, we have found through this review that the 90% threshold paired with NNMF provides meaningful insight for measuring stroke motor control during walking. This is corroborated by simulation work that shows modules determined with this VAF threshold in healthy individuals; each demonstrates different biomechanical functions and produces well-coordinated walking [15, 16, 25, 39].

The number of muscles measured during a task may also impact module number selection. Upper extremity simulation work by Steele et al. [40] reported that when fewer muscle groups were included in an NNMF analysis a lower VAF was found in comparison to an analysis with all possible muscle groups. Unfortunately, limitations of surface EMG methodology prevent the measurement of every muscle that contributes to walking. To address this concern, Steele et al. [40] recommended selecting dominant muscles for task completion, or those with the largest force production. They also report that measuring more than 7 muscles, on average, accounted for 90% of the VAF in comparison to the full muscle set of 30 [40]. Through our review, we found that muscle coordination during healthy and hemiparetic walking can be evaluated by assessing modules formed from the following set of eight muscles during self-selected steady state walking: tibialis anterior, soleus, medial gastrocnemius, rectus femoris, vastus medialis, medial and lateral hamstrings, and gluteus medius. These eight muscles are strong contributors to walking and are thought to provide a majority of the force production to accomplish walking tasks satisfying recommendations from Steele et al. [40]. In addition, these eight muscles have shown the ability to account for over 90% of the VAF in healthy individuals and are supported by simulation analysis to show that 4 modules determined from this set are sufficient for accomplishing the majority of the biomechanical functions necessary to produce walking in healthy individuals [15, 39]. While more modules may be able to be identified using a greater number of muscles [15, 27, 34], 4 modules identified by a 90% VAF selection criteria were found to provide sufficient differentiation between walking in healthy individuals and those poststroke.

There is still a need to determine the origin of muscle coactivation patterns that are present in the modules. The literature reveals many different interpretations of what modules represent at the level of neural mechanisms [41–43]. Views range from the coordinated muscle activity represented by modules being the direct result of “hardwired” central nervous system pathways to modules only being reflective of the biomechanical constraints induced by the task [44, 45]. Research investigating these neural mechanisms is important and has the potential to yield powerful insights into motor control [10, 41, 45], regardless of whether modules are fundamental nervous system building blocks or nervous system solutions to specific motor tasks. Nevertheless, upon synthesis of the research in this review, it becomes apparent that modules provide a quantitative framework for measuring ability to move progressively more independent from mass flexion and extension coactivation towards independent control of muscle group activation as required for walking. This appears to be especially true for individuals poststroke who use fewer modules when walking with higher amounts of muscle coactivation [14, 25, 26]. While there are important questions that remain regarding the physiological basis for modules, we believe that modules provide a valuable quantitative analysis technique for testing theories of poststroke motor control.

Typically, there is a reduction in modules in the paretic leg by an individual during walking after a stroke [14, 25–34] and multiple studies correlated use of fewer modules with poor walking performance [14, 25, 26, 32–34]. Similar relationships exist between the use of fewer module and rehabilitation outcome measures. While the Fugl-Meyer Assessment is currently the gold standard for measuring motor control impairments in individuals poststroke [8], a module-based analysis of muscle coordination may be superior for evaluating a functional mobility construct of motor control. This is supported by Bowden et al.'s [26] report of a stronger correlation between module number and several biomechanical measures of walking than the Fugl-Meyer and Barroso et al.'s [34] finding that VAF in the nonparetic limb from a module analysis was superior to the Fugl-Meyer in predicting walking speed. Additional evidence for a functional construct is supported by Ferrante et al.'s [28] report that the number of modules may be more responsive to rehabilitation than the Fugl-Meyer Assessment for walking in individuals poststroke.

There is also evidence to suggest that module composition or control may provide finer grained understanding of the neural impairments underlying the differences between modules [14, 32]. This was supported by Routson et al. [32] who found that individuals who use 4 modules prerehabilitation improve the timing of module 2 in response to rehabilitation. Theoretically, improvements in timing of module 2 (ankle plantarflexors) should result in improved timing of ankle propulsion for swing initiation. The ability to improve timing of a module (i.e., module control) without increasing the number of modules used suggests that changes in module control may also be a marker of improvements in muscle coordination in addition to using more modules.

The importance of understanding changes in module number or control is important because both appear to respond to rehabilitation. However, treatment response may be dependent on the initiation of rehabilitation in response to time since initial injury or intervention type. Ferrante et al. [28] and Routson et al. [32] demonstrated improvements in module use with interventions for improving motor control that were task specific using treadmill training. In contrast, Hashiguchi et al. [30] found that individuals with subacute stroke did not improve module number with inpatient rehabilitation that was not gait training focused. Study design may account for this finding. For example, there was a smaller sample size and participants could use assistive devices during EMG recordings. However, the inpatient rehabilitation program was focused on activities of daily living and functional mobility rather than task-specific exercises meant to improve coordination, likely contributing to the lack of changes in module use. That modules appear to respond to interventions provide more evidence of the strength of a module-based analysis of muscle coordination to quantify motor recovery in individual poststroke.

### 4.1. Future Research

Increased effort to evaluate modules across a larger representation of the stroke population will provide understanding of how module expression varies across severity, changes with spontaneous recovery, and responds to rehabilitation. Emphasis should be placed on the modules' potential to provide specific targets for designing treatments through better understanding of the specific neural impairments they represent and the resulting biomechanical consequences. Future studies should continue to evaluate the impact of using module-driven interventions to enhance motor control.

### 4.2. A Recommended Approach for Module-Based Analyses of Poststroke Walking

Based on the synthesized literature, we present the following approach for a module-based analysis of walking poststroke. We recommend applying NNMF to EMG from the following eight muscle set: tibialis anterior, soleus, medial gastrocnemius, rectus femoris, vastus medialis, medial and lateral hamstrings, and gluteus medius during steady state walking with a preselected 90% VAF threshold to identify modules that will provide a quantifiable and insightful way to characterize healthy and poststroke muscle coordination during walking. Additionally, this module-based approach will be able to distinguish one's locomotor complexity or ability to move away from mass flexion and extension muscle coactivation during walking. Synthesizing the available evidence, modules appear to provide quantitative insight that outperforms current rehabilitation outcomes such as the Fugl-Meyer Assessment for quantifying motor control during walking tasks. Specifically, we suggest the consideration of a module-based framework for measuring performance changes in muscle coordination during poststroke walking as a result of rehabilitation treatments.

## 5. Conclusion

The results synthesized in this review reveal that module-based analyses seem very well suited for providing a motor control framework to answer important research and clinical questions about hemiparetic walking. Specifically, this review synthesized the following evidence: (1) decreases in number and quality of modules after stroke are well correlated with stroke severity; (2) the number of modules outperforms the current gold standard assessment of motor impairment in capturing walking impairments; and (3) the quality and number of modules can be changed by intensive task-specific rehabilitation that improves walking function. Regardless of whether future research shows that modules reflect hard-wired neural circuitry or are more reflective of output organized to perform the specific tasks of walking based on biomechanical constraints, a module-based analysis using eight leg muscles with an NNMF-based factorization scheme has been shown to produce great insight into poststroke motor control based on muscle coordination during walking. Since individuals poststroke use a reduced number of modules during the gait cycle compared to healthy controls and the number of modules individuals use correlates better with biomechanical walking performance than the Fugl-Meyer Assessment, we conclude that module-based analyses quantitatively measure the ability to move away from mass flexion-extension coactivation towards more differentiated control of muscle groups during walking. Additionally, because the number and quality (closer agreement with the typical number or timing patterns shown by healthy individuals) of modules appear to increase with focused task-specific rehabilitation of the paretic leg, module-based analyses appear to quantify improvement or progression of motor control that likely reflects underlying changes in neuroplasticity. Future work should aim at increasing our understanding of how module composition and control, as well as commonly seen impaired module patterns, relate to biomechanical function and respond to intervention in order to determine whether they can provide measures to more accurately target therapies addressing muscle coordination during walking.

## Figures and Tables

**Table 1:** Search terms.

MEDLINE (Completed via PubMed)^∗^
Search 1: (((((("Cerebrovascular Disorders"[Mesh]) OR stroke OR "brain infarct^∗^" OR CVA OR "cerebrovascular accident")) AND (EMG OR electromyography OR motor OR locomot^∗^ OR biomechanic^∗^ OR movement)))) AND (modul^∗^[Title] OR mode[Title] OR pattern[Title] OR synergy[Title]) AND ("last 11 years"[PDat] AND Humans[Mesh] AND English[lang])
Search 2: ("Cerebrovascular Disorders"[Mesh]) AND ((modul^∗^ OR mode OR pattern OR synergy OR EMG OR electromyography OR motor OR locomot^∗^ OR biomechanic^∗^)) AND ("Movement Disorders"[Mesh]) AND ("last 11 years"[PDat] AND Humans[Mesh] AND English[lang])
SCOPUS^∗^
( TITLE-ABS-KEY (emg OR electromyography OR motor OR locomot^∗^ OR biomechanic^∗^ OR movement) AND TITLE ( modul^∗^ OR mode OR pattern OR synergy ) AND TITLE ("Cerebrovascular Disorders" OR stroke OR "brain infarct^∗^" OR cva OR "cerebrovascular accident" AND (LIMIT-TO (LANGUAGE, "English")) AND (LIMIT-TO (PUBYEAR, 2018) OR LIMIT-TO (PUBYEAR, 2017) OR LIMIT-TO (PUBYEAR, 2016) OR LIMIT-TO (PUBYEAR, 2015) OR LIMIT-TO (PUBYEAR, 2014) OR LIMIT-TO (PUBYEAR, 2013) OR LIMIT-TO (PUBYEAR, 2012) OR LIMIT-TO (PUBYEAR, 2011) OR LIMIT-TO ( PUBYEAR, 2010) OR LIMIT-TO (PUBYEAR, 2009) OR LIMIT-TO (PUBYEAR, 2008 ) OR LIMIT-TO ( PUBYEAR, 2007))
CINAHL^∗^
(MH "Stroke+") OR (MH "Cerebral Ischemia+") AND modul^∗^ OR pattern OR synergy OR mode AND EMG OR electromyography OR motor OR locomot^∗^ OR biomechanic^∗^ OR movement
Limiters - Published Date: 20070101-2018; English Language; Exclude MEDLINE records
Search modes - Boolean/Phrase
*No additional articles were found using PEDro or OT Seeker using key search terms related to stroke, EMG, modules or motor synergies*

^∗^All searches were completed on April 24, 2018.

**Table 2:** Study characteristics.

Author, year	Design	Demographics (individuals poststroke)	Demographics (healthy controls)	Muscle sets for EMG observation	Factorization technique
Allen et al. 2013 [25]^∗^	Cross-sectional	*n* = 11 Sex: 7 males, 4 females; age: 62.2 ± 11.7; time since stroke: 3.5 ± 2.7 years	*n* = 14 Sex: 2 males, 12 females; age: 63.1 ± 9.1	TA, SO, MG, VM, RF, MH, LH, GM	NNMF

Barroso et al. 2017 [34]	Cross-sectional	*n* = 9 Sex: 6 males, 3 females; age: 53 ± 11.05; time since stroke: 75.6 ± 65.9 months	None	TA, SO, MG, VL, RF, BF, GM, GMax, TFL, ADL, ES	NNMF

Bowden et al. 2010 [26]^∗^	Cross-sectional	*n* = 55 Sex: 35 males, 20 females; age: 59.5 ± 11.7; time since stroke: NR	*n* = 20 Sex: 4 males, 16 females; age: 65.5 ± 9.8	TA, SO, MG, VM, RF, MH, LH, GM	NNMF

Clark et al. 2010 [14]^∗^	Cross-sectional	*n* = 55 Sex: 35 males, 20 females; age: 59.5 ± 11.7; time since stroke: 57.8 ± 64.8 months	*n* = 20 Sex: 4 males, 16 females; age: 65.5 ± 9.8	TA, SO, MG, VM, RF, MH, LH, GM	NNMF

Coscia et al. 2015 [27]	Cross-sectional	*n* = 12 Sex: 9 males, 3 females; age: 58.53 ± 16.37; time since stroke: 54.6 ± 56.2 months	*n* = 10 Sex: 9 males, 3 females; age: 63.3 ± 3.1	PERL, TA, SOL, LG, RF, VM, BF, ST, ADL, TFL, GM, GMax	Factor analysis

Ferrante et al. 2016 [28]	Prepost experimental	*n* = 2 Sex: 2 males, 0 female; age: 67, 64; time since stroke: 11 years, 9 months	*n* = 13 Sex: 7 males, 6 females; age: 24.8 ± 1.3	TA, MG, MH, LH, VM, RF, GMax	NNMF

Gizzi et al. 2011 [29]	Cross-sectional	*n* = 10 Sex: 8 males, 2 females; age: 45.9 ± 16.5; time since stroke: 12 ± 5 weeks	*n* = 10 Sex: 7 males, 3 females; age: 42.4 ± 14.5	TA, MG, SOL, VL, RF, BF, GMax, RA, ES, LD, BB, TB, AD, UT, ST, SPL	NNMF

Hashiguchi et al. 2016 [30]	Prepost experimental	*n* = 13 Sex: 10 males, 3 females; age: 58.8 ± 13.2; time since stroke: 66.8 ± 24.2 days	None	TA, LG, SO, RF, VM, BF, ST, GM	NNMF

Kautz et al. 2011 [31]^∗^	Cross-sectional	*n* = 56 Sex: 36 males, 20 females; age: 61.0 ± 12.3; time since stroke: 5.1 ± 5.6 years	*n* = 17 Sex: 2 males, 15 females; age: 65.1 ± 10.4	TA, SO, MG, VM, RF, MH, LH, GM	NNMF

Routson et al. 2013 [32]^∗^	Prepost experimental	*n* = 22 Sex: 15 males, 7 females; age: 57.3 ± 13.2; time since stroke: 19 ± 13 months	None	TA, SO, MG, VM, RF, MH, LH, GM	NNMF

Routson et al. 2014 [33]^∗^	Prepost experimental	*n* = 27 Sex: 18 males, 9 females; age: 60.15 ± 12.08; time since stroke: >6 months	*n* = 17 Sex: 9 males, 8 females; age: 54.18 ± 8.33	TA, SO, MG, VM, RF, MH, LH, GM	NNMF

TA: tibialis anterior; PERL: peroneus longus; SO: soleus; MG: medial gastrocnemius; LG: lateral gastrocnemius; VM: vastus medialis; VL: vastus lateralis; RF: rectus femoris; TFL: tensor fasciae latae; BFlh: biceps femoris long head; BFsh: biceps femoris short head; ST: semitendinosus; ADL: adductor longus; MH: medial hamstrings; LH: lateral hamstrings; GM: gluteus medius; GMax: gluteus maximus; RA: rectus abdominis; ES: erector spinae; LD: latissimus dorsi; BB: biceps brachii; TB: triceps brachii; AD: anterior deltoid; UT: upper trapezius; ST: sternocleidomastoid; SPL: splenius capitis; NR: not reported; NNMF: nonnegative matrix factorization; PCA: principal components analysis. ∗ indicates the publication came from the same research group and used a subset of the same sample.

**Table 3:** Study findings (cross-sectional designs).

Author, year	Study purpose	Primary findings	Findings related to module number, composition and control, gait, and rehabilitation outcomes
Allen et al. 2013 [25]	Determines biomechanical functions for modules used during poststroke walking using EMG data for simulations	Common merging patterns category A (modules 1 with 2) and category B (module 1 with 4) are correlated with common gait impairments seen in individuals poststroke	Category A: (1) Reduced propulsion during push off due to increased hamstring activity (2) Reduced limb swing (3) Reduced mediolateral stability during stance
			Category B: (1) Reduced propulsion during push off due to premature plantarflexion (2) Reduced limb swing (3) Reduced body support during initial stance

Barroso et al. 2017 [34]	Determines if gait mechanics and modules are better predictors of walking performance than current clinical assessments (Fugl-Meyer)	Individuals poststroke used a range of 2–5 modules. There was a significant difference between mean VAF between the paretic and nonparetic limbs for individuals with 3, 4, or 5 modules (*p* < 0.018).	Stepwise multiple linear regression: Overground gait speed = 7.785–0.48 (time of peak nonparetic knee flexion) − 3.672 (nonparetic VAF with 4 modules) *R*^2^ = 0.885, *p* = 0.001

Bowden et al. 2010 [26]	Determines the relationship of module use with gait mechanics and rehabilitation outcome measures	Module number was found to have higher correlations with functional outcomes and gait kinematics than the FM-LE or FMS	Gait kinematic correlations with module number Pp *r* = −0.389, *p* = 0.023 PSR *r* = −0.558, *p* = 0.001 PPS *r* = −0.398, *p* = 0.020
			Functional outcome correlations with module number Speed *r* = 0.451, *p* = 0.008 BBT *r* = 0.504, *p* = 0.003 DGI *r* = 0.545, *p* = 0.002

Clark et al. 2010 [14]	Determines if module use differences between healthy individuals and those poststroke are associated with walking performance	Number of modules used in the paretic limb during walking predicted performance	58% of participants required four modules 45% of participants required two modules 36% of participants required three modules
			Of 3 module participants, 8/19 demonstrated a merging of modules 1 and 2 (category A) and 7/19 demonstrated a merging of modules 1 and 4 (category B).
			Gait correlations with module use: SS speed *p* = 0.5, *p* = 0.0002 Speed modulation *p* = 0.47, *p* = 0.0008 Propulsive asymmetry *p* = −0.28, *p* = 0.04 Step length asymmetry *p* = −0.32, *p* = 0.02

Coscia et al. 2015 [27]	Evaluates relationships between gait asymmetries and changes in modules	Factor analysis resulted in a range of modules (3–5) to explain the gait cycle variance. The authors selected 3 modules as their maximum value which explained 75% of the variance.	In participants poststroke, module 1 explained a greater degree of variance than in healthy controls
			Changes in speed did not alter the number of modules, but did have a significant effect on weight coefficients for module 1 (*p* = 0.003) and module 2 (*p* = 0.027) between a stroke participant's paretic and nonparetic legs.

Gizzi et al. 2011 [29]	Determines if the number of modules is similar in individuals with subacute stroke (≤20 weeks) and if the inclusion of more muscles will change the number of modules	The number of modules was consistent with the previous findings in chronic stroke ranging from 2–4. The inclusion of upper extremity and trunk musculature did not change the number of modules.	Module number was equivalent using NNMF for the set of 16 muscles and set of 7 lower extremity only muscles.
			Timing patterns for each module did not differ between limb-affected side (*r* = 0.74); unaffected side of patients, (*r* = 0.75); or healthy controls (*r* = 0.78, *p* = 0.05)

Kautz et al. 2011 [31]	Determines if there is a difference between TM and OG walking on module assignment	There was no difference between the numbers of modules assigned for individuals walking on the TM or OG despite differences in speed.	Module number explained greater than 90% variance for participants and controls for SS on TM (91.9% ± 4.1%, 93.5% ± 3.5%) OG (95.2% ± 2.8%, 97.5% ± 1.0%)
			Hemiparetic participants walked slower on the TM (TM, 0.38 versus OG, 0.58 m/s; *p* < 0.001), with increased cadence (TM, 84.9 versus OG, 77.6 steps/min, *p* = 0.04) and decreased stride length (TM, 0.52 versus OG, 0.85 m; *p* < 0.0001).

Routson et al. 2014 [33]	Determines if task variation results in module changes after a steady-state is reached	Varying SS speed walking conditions with maximum cadence, maximum step length, and maximum step height did not change the number of modules used.	In healthy controls, all tasks demonstrated 4 modules (*p* = 0.78).
			Number of modules correlated to a reduced ability to change speed (*p* < 0.0001), cadence (*p* < 0.0001), step height (*p* < 0.0001), step length (*p* < 0.0001)

PC: principal component; FM-LE: Fugl-Meyer lower extremity; FMS: Fugl-Meyer synergy; BBT: Berg Balance Test; DGI: dynamic gait index; Pp: paretic propulsion; PSR: paretic step ratio; PPS: paretic preswing; SS: self-selected; FC: fastest comfortable; OG: over ground; TM: treadmill.

**Table 4:** Study findings (prepost experimental designs).

Author, year	Intervention	Changes in module number	Changes in module composition and control	Changes in gait outcomes	Changes in rehabilitation measures
Ferrante et al. 2016 [28]	FES-supported treadmill walking for 30 minutes, 3 times/week for 4 weeks	Both subjects (S1, S2) increased module number from 3 to 4.	S1: initial merging of modules 1 and 4 S2: initial merging of modules 3 and 4	Gait speed (pre/post) S1: 0.43/0.88 m/s S2: 0.38/0.68 m/s GRC (change score) S1: +4 S2: +2 Cadence (pre/post) S1: 0.98/1.01 strides/s S2: 0.81/0.80 strides/s	S1 (pre/post) Mini Best test (17/22) FIM motor (78/78) S2 (pre/post) Mini Best test (21/23) FIM motor (85/85)

Hashiguchi et al. 2016 [30]	1 month of inpatient rehabilitation (gait, balance and task-specific training), 60 min/day, 5 days/week	No significant change in module number (*p* = 0.73)	Paretic muscle strength index and ankle range of motion correlated with the merging index Strength *β* −0.558 (−1.26, −0.17) *p* < 0.05 Range of ankle *β* −0.481 (−1.16, −0.07) *p* < 0.05 BI correlated with the fractionalization index *β* 0.577 (0.15, 4.84) *p* < 0.05	Gait speed significantly improved postrehabilitation (*p* < 0.01).	Paretic muscle strength index improved significantly postrehabilitation (*p* < 0.05). BI, BBT, and TUG all had significant improvement postrehabilitation.

Routson et al. 2013 [32]	A 12-week, 36 session locomotor training program with body weight support and manual assistance	All subjects attained 4 modules postrehabilitation (*n* = 22)	Individuals with 4 modules pre- and postrehabilitation improved the timing of module 2 to match healthy controls (*p* < 0.65). Module 2 composition differences were significantly different from healthy controls in individuals with 3 modules prerehabilitation (*p* < 0.001).	Significant improvements in gait speed: (*p* = 0.011); preswing leg angle: (*p* = 0.044) Nonsignificant improvements in PP: (*p* = 0.1121); PSR: (*p* = 0.6904) Subjects with 3 modules prerehabilitation had increased step length, propulsion asymmetry, and reduced SS gait speed and preswing leg angle than controls (*p* < 0.05)	None reported

FES: functional electric stimulation; GRC: global rating change; BBT: Berg Balance Test; BI: barthel index; TUG: timed up and go; PP: paretic propulsion; PSR: paretic step ratio; SS: self-selected; FC: fastest comfortable; Paretic muscle strength index (N·m/kg): sum of hip flexor, knee extensor, knee flexor, ankle dorsiflexor, and ankle plantar flexor.

**Table 5:** Modified downs and black scores.

Article	Reporting							External validity		Internal validity: bias		Internal validity: confounding				Total	Percent
	1. Is the hypothesis or objective clearly stated?	2. Are the main outcomes to be measured clearly described?	3. Are the characteristics of the participants clearly described?	5. Are the distributions of principle confounders clearly described?	6. Are the main findings of the study clearly described?	7. Estimates of the random variability in the data for the main outcome?	10. Have actual probability values been reported?	11. Were the subjects asked representative of the entire population?	12. Were those subjects used representative of the entire population?	16. Were any of the results based on “data dredging”?	18. Were the statistical tests used to assess main outcome measures appropriate?	20. Were the main outcome measures used accurate? (valid and reliable?)	21. Were cases and controls from the same population?	22. Were the cases and controls recruited over the same period of time?	25. Was there adequate adjustment for confounding in the analysis?		
Allen et al. [25]	1	1	1	1	1	1	NA	0	0	1	NA	1	0	1	NA	9	69%
Barroso et al. [34]	1	1	1	1	1	1	1	0	0	1	1	1	NA	NA	1	12	75%
Bowden et al. [26]	1	1	1	1	1	1	1	0	0	1	1	1	0	1	1	12	75%
Clark et al. [14]	1	1	1	2	1	1	1	0	0	1	1	1	0	1	1	13	81%
Coscia et al. [27]	1	1	1	1	1	0	1	0	0	1	1	1	0	1	1	11	69%
Ferrante et al. [28]	1	1	1	1	1	0	0	0	0	1	NA	1	0	1	1	9	60%
Gizzi et al. [29]	1	1	1	1	1	1	0	0	0	1	1	1	0	1	1	11	69%
Hashiguchi et al. [30]	1	1	1	1	1	1	0	0	0	1	1	1	0	1	1	12	75%
Kautz et al. [31]	1	1	1	1	1	1	1	0	0	1	1	1	0	1	1	13	81%
Routson et al. [32]	1	1	1	1	1	1	1	0	0	1	1	1	0	1	1	13	81%
Routson et al. [33]	1	1	1	1	1	1	1	0	0	1	1	1	0	1	1	13	81%
